# An Investigation Into the Orthoptist Experience of Instilling Eye Drops in Children Attending the Eye Clinic

**DOI:** 10.22599/bioj.314

**Published:** 2024-08-14

**Authors:** Hanish Chauhan

**Affiliations:** 1 University Hospitals of Leicester NHS Trust, UK

**Keywords:** orthoptist, children, eye drops, instilling, eye clinic

## Abstract

**Purpose:**

Eye drops instillation in children is a fundamental part of accurately examining a child’s eyes. Unfortunately eye drops can be a distressing experience for children, parents/guardians and orthoptists. The purpose of this research is to focus on the experiences of orthoptists and delve deeper into their views and explore if improvements can be made.

**Methods:**

This was a Qualitative study involving semi-structured interviews with 8 registered and currently practicing orthoptists in the UK. The interviews were undertaken online via Microsoft TEAMS. Thematic analysis was carried out for the purposes of data analysis.

**Results:**

3 major themes were identified (1) how orthoptist frame instilling eye drops, (2) techniques to address challenges, and (3) improvements to eye drops instillation process. Orthoptists were aware that instilling eye drops in children brought specific challenges such as distress and resistance; however they saw it as an essential part of their job. Orthoptists understood their role within a wider team which delivered eye care to children effectively and that there was a division of they believed that. Orthoptists were aware that the eye drops may cause some distress, however this did not affect compliance with treatment such as wearing glasses and/or a patch. Orthoptists believed verbal and non-verbal communication with the child was essential. Help was sought from parents or colleagues for physical restraint if required. Orthoptists suggested adapting to children with additional needs and giving out eye drops to parents/carers to instill at home if dilation in the eye clinic became difficult. They suggested improvements such as assistance from play specialists, developing a pre-procedural information video, practise as a student, the study of the medical exemptions module and the potential of using eye sprays instead of eye drops.

**Conclusion:**

The study reiterates the importance of verbal and non-verbal communication. The results may facilitate recommendations for change such as encouraging the study of medical exemptions and help support a case for play specialist support regularly, and the encouragement to develop a pre-procedural information video to improve quality of care. This is currently inconsistent across different Trusts in the UK. The study could result in improvements to current practise and influence other fields of medicine such as blood tests and MRI scans in children. The study also recommends further studies to investigate the parental perspective of instillation of eye drops in their child’s eyes when they attend the eye clinic.

## Introduction

Cycloplegic eye drops are essential for accurately examining children referred for or diagnosed with amblyopia, refractive error and/or any other ocular pathology. UK orthoptists have instilled eye drops in children’s eyes for many years. Eye drops are usually administered under a PGD (patient group direction) enabling orthoptists to use eye drops in an identified clinical situation (NHS England 2024) without prescription. Unfortunately, eye drops can be a distressing experience for children, parents/guardians and orthoptists; the stress can have a negative impact on the remainder of the eye examination because the child may become uncooperative for the refraction or fundus examination. Distress in children can present itself in a variety of ways such as crying, screaming, covering eyes, burying head into parents and panicking. A negative experience from instillation of eye drops may cause fear and a loss of confidence, which can manifest into a poor relationship between the orthoptist, child and parents and, thus, potentially affect treatment compliance.

Several studies have explored distress caused to children when receiving eye drops. Chafai et al. (2006), Syrimi et al. (2013) and Vagge et al. (2020) compare the use of eye sprays versus eye drops and the levels of distress caused in children. Bray et al. (2016), Kolk et al. (1999) and Pilon et al. (2020) researched levels of distress in children during other hospital procedures, such as blood tests and venepuncture. However, there are minimal studies on the clinician perspective of undertaking an invasive procedure in children in general. There are currently no studies exploring the experiences of orthoptists when instilling eye drops in children despite it being undertaken on a daily basis. The purpose of this research is to focus on the experiences of orthoptists and delve deeper into their views.

There are currently no studies which explore the use of distraction techniques during instillation of eye drops in children. This research will ask orthoptists how they respond to the challenges of a distressed child and the repertoire of practises and techniques they deploy to instil eye drops successfully.

### Aims

The aims of the study are:

- To explore the experiences and views of orthoptists regarding encounters in which they instil eye drops in a child’s eyes.
- To explore if improvements can be made or specific techniques can be introduced to improve the experience when instilling eye drops.

## Methodology

This was a qualitative study using semi-structured interviews on eight participants.

### Participants, sampling and recruitment

Before study commencement, ethical approval was received from the Health Research Authority and De Montfort University. A convenience sample of eight participants was recruited for the study through a professional network. Current colleagues were asked to contact previous colleagues from another hospital Trust to see if they were interested in taking part in a study. The contact details were then shared with the researcher and further information was sent to the participant. Whilst this did not allow generalisability to the wider population of orthoptists, it offered an insight into everyday experiences of orthoptists within an interpretivist study.

The inclusion criteria were as follows:

- Current practising orthoptists
- Registration with the Health and Care Professions Council
- Regularly instil eye drops in children attending the eye clinic.

The exclusion criteria were:

- Individuals not currently practising as an orthoptist
- Unregistered orthoptists; although they may have previous relevant experience of instilling eye drops, the study sought to find recent experiences.
- Staff from the NHS hospital Trust where the researcher has line management responsibilities. This could have resulted in interview bias and prospective participants feeling obligated to participate.

### Data collection and analysis

A topic guide (Appendix 1) was used to identify key issues to outline areas of questioning. The interview questions were piloted with two orthoptists to ensure relevant data was captured. The topic guide began with questions around the background experience of the participants and their years of experience; this was to make them feel settled, gauge how long they had been working and whether they had worked in different hospitals. This helped phrase some of the upcoming questions appropriately and delve into their experiences.

All interviews with orthoptists were carried out online via Microsoft TEAMS. An invitation link was sent to the participant via email and the interview took place in a pre-booked private study room at De Montfort University using a university laptop. Online interviews consisted of a combination of open-ended questions, follow-up questions and prompts. All interviews were audio recorded with no video. The semi-structured interviews lasted between 20 to 35 minutes and were transcribed into a written form and all data was stored securely online on the university’s Microsoft OneDrive. Each transcript was allocated a label from P1 to P8 to ensure the transcripts remained anonymous.

Thematic analysis was applied to the written transcripts, conducted in six phases, following methodology by Braun and Clarke (2022).

## Results

### Participants

The participants’ levels of experience working as an orthoptist ranged from 2–14 years, and all were from different NHS Trusts across the United Kingdom. 87.5% of participants had worked at different hospital Trusts previously, whilst 25% of participants had also worked abroad in Australia. Participants consisted of seven females and one male; this is typical of the orthoptic profession, which is female dominated. This is evident in the recent HCPC diversity data report which found 87% of orthoptists in the United Kingdom were female (HCPC diversity data report 2023).

### Data analysis

The initial thematic map is shown in Appendix 2 and the final thematic map is shown in Appendix 3, which includes the themes and the sub-themes which were identified following the final analysis. The over-arching theme identified was ‘Eye drops instillation in children requires resourcefulness in the execution of the procedure as part of the orthoptic role’ and this was followed by the 3 major themes below:

1. How the orthoptist frames instilling eye drops
2. Techniques to address challenges
3. Improvements to eye drops instillation process

### How the orthoptists Frames instilling eye drops

This was an important first major theme, which included the subthemes mentioned in Table 1. All participants believed that the eye drops were an important aspect of their role, participants viewed it as ‘part of the job’ as evident in Table 1 and seemed to take it in their stride. P7 alludes to the importance of eye drops in enabling others providing eye care to do their job effectively for the good of the child’s eye health. This was an important finding because it conceptualised how orthoptists viewed their role in delivering eye care as part of a ‘division of labour’ within a department usually consisting of several other eye care professionals, such as nurses, healthcare assistants, optometrists and ophthalmologists. This ‘division of labour’ was mentioned by most participants, and was often accompanied by the observation that others, including optometrists and doctors, typically preferred eye drops to be instilled by someone other than themselves partly to protect the relationships they fostered with the child. The main reason stated for this was to minimise poor compliance during examination of the eyes because the child would usually see the optometrist or doctor after the orthoptist. Whilst most participants were happy to accept that there was a division of labour in the eye care of children, P3 felt that orthoptists may try to avoid it and there could be some reluctance but also states that it was down to orthoptists to instil eye drops.

**Table 1:** Quotes that illustrate how orthoptists frame instilling eye drops.


Part of the job	*‘I also think it’s a very important part of our job and it enables thorough examinations to take part’. (P1)*

	*‘We’re here to help the patient, and we know that eye drops aren’t the nicest of things to experience, but they are necessary evil to make sure we’re giving the patient the best care’. (P7)*

Division of labour	*‘In order to do our jobs, in order to let the doctors do their jobs, in order to let the optometrists do their jobs, we have to put eye drops in’. (P7)*

	*‘I know our optometry colleagues feel quite strongly that because they’ll be seeing the patients after we have put the drops in, they do not like to instil the drops themselves’. (P1)*

	*‘Because they think they’ve lost any potential co-op umm with the patient and but I guess as orthoptists because we put the eye drops in and then very rarely we see them again afterwards’. (P1)*

	*‘The optometry team wouldn’t do it, and they would ask us to do it, because they would say, we then have to do the test they might not comply for us’. (P2)*

	*‘We seem to be the sort of bad guys I think. ‘cause if people have got to do an assessment after us, they don’t want to be the ones to put the drops in. Whereas because at this point we’ve sort of finished our assessment, it’s down to us to sort of put them in. So yeah, I think there’s a little bit of hesitancy to be the actual one to do it’. (P3)*

Challenging task	*‘The standard comments are, oh, we don’t want eye drops as soon as they walk into the clinical room’. (P7)*

	*‘When the child has become so distressed that even if you did instil them that they’re going to be so distressed that you’re not going to get the refraction’. (P5)*

	*‘I’ve been nipped, scratched, kicked, spat on at one point’. (P4)*

	*‘If the legs got a little bit out of control, I’ve had a bit of like a push, but it’s not hurt me as such’. (P4)*

Treatment unaffected	*‘Well, definitely for occlusion, it’s more of like you go home and do it, yeah. It’s a complete separate thing. So I think most of the time the kids don’t really associate. I mean, if they don’t like the patch, it’s because they don’t like the patch rather than the like, you know, having to have eye drops or anything’. (P4)* *‘I suppose a different thing. I don’t think it would kind of impact on it like that I think, especially as for the patching at home’. (P8)*


Whilst all participants saw instilling eye drops as an essential and an unavoidable aspect of the job, they all perceived the administration of eye drops as posing practical problems. The fear experienced by children was a recurrent theme in orthoptists’ accounts and some referred to fear arising from past experiences which made them frightened to return to the eye clinic. This made it a ‘challenging task’; therefore, this subtheme was created to capture this. Participants recognised that with many children, the instillation of eye drops will cause some form of distress . However, the challenges of instilling them did not outweigh the benefits. Regardless of causing distress and difficulties to children, the eye drops were framed as necessary and part of the role. Another factor that made it challenging included instances of being physically hurt by children during attempts to administer eye drops. P5 summarises she had been hurt whilst trying to instil eye drops; however, P5 and P2 also indicated that it has never affected them long-term and did not cause an impact on them physically or mentally.

Whilst the instillation of eye drops in children was seen as a ‘challenging task’, the orthoptists believed eye drops instillation did not affect compliance with treatment issued by them, such as patching or the use of eyeglasses. They felt children did not associate eye drops in clinic with treatment at home. This was stated by all participants as indicated below and captured by the subtheme ‘treatment unaffected’.

### Techniques to address challenges

This second major theme included the subthemes stated in Table 2. The most frequently mentioned technique for successful administration was effective communication, which included verbal and non-verbal techniques. Participants spoke about the challenges of dealing with a frightened or anxious child and how verbal ‘effective communication’ was key in trying to soothe the child by promising to instil eye drops as quickly as possible. Participants believed instilling eye drops as speedily as possible was essential to reduce any distress caused to the child or parent. Orthoptists chose words carefully and avoided specific words which were thought to trigger a disruptive reaction such as ‘sting’ as described in Table 2. Participants preferred to use gentler words with positive associations such as ‘magic’, ‘tingly’ or ‘sparkly’. Communication included non-verbal gestures such as demonstrating the eye drops on the child’s hands allowing them to see the eye drops as non-threatening and something familiar, such as water. For communication to be effective it was tailored to the age and understanding of the child as P1 and P6 states. The purpose of this was to calm the child who was frightened and pre-empt fear if possible, as well as, where suitable, to appeal to the child’s reason and to conceal the difficulties or distract them. Parents were alerted to the difficulties and the conversations included the side effects of eye drops and the expected reaction from the child, so the parents had full understanding. Participants believed it was especially important to adapt to the needs of the child if they had additional needs, such as autism, e.g., to explain the process in more depth so the child knew what to expect and could prepare for it mentally.

**Table 2:** Quotes that illustrate techniques to address challenges.


Effective communication	*‘So I usually calm them down and I say okay, we’ll try and do it ask quick as we can’. (P2)*

	*‘I don’t tell them it’ll sting, but something that might feel a bit funny and just to blink a lot afterwards and that funny feeling will go away’. (P2)*

	*‘We need to see put some magic water into your eyes and make them nice and big so we can have a look to see if you need any glasses and just to make sure your eyes are nice and strong and healthy and always say it’ll feel a bit cold or a bit tingly or it’ll feel a bit funny’. (P2)*

	*‘So sometimes I let them see, like I’ll put a drop on their hands so that they can see like the consistency of what it is’. (P5)*

	*‘We’ll say that we’re going to look inside your eye to see if the machines are working OK, that sort of thing. So I guess just trying to tailor it to the age of the child, what their possible interest might be’. (P1)*

	*‘If they’re old enough to understand that they’re going to have eye drops in, and what the purpose is, I’ll explain to the child that we need to put a little drop of water with each eye, and it does feel a bit funny’. (P6)*

	*‘I’ll try and discuss it with the parent a little bit more sort of subtly so the child isn’t necessarily aware of what’s coming up, because I don’t want them to start getting worried before I even got close to them sort of thing and want them to gain consent with the parent to do so’. (P3)*

Adapt to additional needs	*‘So if we know they’ve got additional needs, we tend to take them into their own room, it can get quite noisy so make sure the child is in a quiet room by themselves and there’s no other distractions’. (P6)*

Help with physical restraint	*‘So the one thing that I find helpful is I kind of get the parent to cradle them’. (P4)*

	*‘I normally say can you pop them up on your lap sort of cradle them like a baby and I normally say put the head in the crease of your elbow and if I think that they’re going to be a kicker, I normally ask them to tuck their legs in between there’s so just in between like their parents legs and cradle them down’. (P5)*

	*‘I would get someone to help come in for both my protection and the child protection so that one of us can perhaps just help to hold the legs down just so they don’t kick’. (P3)*

Home dilation	*‘if I think that it’s not going to happen, it’s just getting a little bit too much for everyone, then I’ll give them that option to take it back at home’. (P4)*

	*‘We do offer sort of pre dilation at home, so we’ll give drops beforehand. And so okay, you’ll just have to come back. We’ll give you the drops to put in home in the home environment’. (P2)*

	*‘Hopefully mean the child is not as distressed about it in the clinic’. (P3)*


All participants indicated that parents were an integral part of helping physically with the process of instillation, and orthoptists asked parents for help regularly. A good explanation and constant communication with parents helped the overall process. The subtheme ‘help with physical restraint’ was created to capture this. Whist this subtheme was about ‘physical restraint’, the orthoptists used terminology such as ‘cradle’, this implied succour and comfort instead of more invasive words such ‘hold down’. At times help was required from colleagues if physical restraint was difficult as quoted in Table 2.

Participants spoke about alternatives to instilling eye drops in the clinic and providing drops to take home for instillation which seemed to have made it easier for the child, parents and orthoptist especially if the child became too distressed or if they closed their eyes too tightly. P2 explains that they use home dilation due to the home environment making the child feel more comfortable. This finding was captured by the subtheme ‘home dilation’. Some of the participants felt home dilation was beneficial because it may reduce distress in the eye clinic and the association between the eye drops and eye clinic was not made.

### Improvements to the eye drop instillation process

The final major theme was ‘improvements to the eye drop instillation process’ it comprised the subthemes as stated in Table 3.

**Table 3:** Quotes that illustrate improvements to the eye drop instillation process.


Study of medical exemptions	*‘My exemptions taught me kind of a good way to do it, where you get the child to close their eye. Obviously, if they’re old enough to understand this and just pop one drop in the corner and then it can get them to blink and it just works its way in’. (P6)*

	*‘you obviously have the medical exemptions module that’s put into the Orthoptic degree now, so I think that the orthoptists who are coming from university now are actually better equipped than I ever was when I left university’. (P7)*

Practise as a student	*‘When I was in training as an orthoptic student, we were instilling eye drops umm so I had that sort of practise then and it was you know very hands on placements. So I think definitely helping students doing it because it’s going to be something that they’re, they’re going to be doing throughout their career’. (P5)*

	*‘I’ve never had an issue with instilling eye drops because when I was on placement many years ago there was a few placements where I needed to help the orthoptist explain the eye drops or help them restrain the child’. (P7)*

	*‘Latest students we’ve had, they’re still not allowed to instil drops and so, uhm, possibly, is there any way, you know, for them to get experience under sort of the guidance of an orthoptist on sort of perhaps their last placement’. (P1)*

Play specialist	*‘Would be great I think if you could have you know a play specialist at every clinic’. (P5)*

	*‘So I didn’t really know how it was gonna go but the play specialist came in. She had like bubbles and toys and she kind of relaxed the child a little bit and then we slowly could do it and it was fine’. (P4)*

	*‘Yeah I think for that certain criteria of children that were discussed, I think for some obviously they don’t need it, but for that that that group of children that need a bit of extra support, I do think that’s helpful’. (P6)*

Information video	*‘We have also got a wee video that come, it’s on our Trust website and it kind of explains. Like it walks through everybody in the department, and it explains to the children and what’s going to happen and that’s for all children’. (P5)*

	*‘There should be some sort of video, maybe something child friendly. I know some parents show their children the Peppa Pig video and I’m going to the eye test and what’s done’. (P2)*

Eye sprays	*‘The eye spray and things like that, I think that would be fantastic that they didn’t even need to open their eyes at all for something’. (P5)*


The medical exemptions module, which was introduced at universities in the last few years, teaches undergraduates and postgraduate orthoptists about eye drops in more depth. It ‘facilitates advanced practise in the area of prescription only and non-prescription exemption listed medicines for orthoptists, by enhancing knowledge of the pharmacokinetics and actions of these specific medicines’ (University of Sheffield: Exemptions, 2023). It also covers instillation techniques. Most participants believed it was beneficial to both students and current orthoptists. P7 had not undertaken the module but felt it would help.

It was apparent that ‘practise as a student’ to instil eye drops would help better prepare future orthoptists for the role and this subtheme was an important finding. Practise as a student was inconsistent and dependent on the placement site according to the participants’ own experiences as students. P5 practised eye drops instillation as a student and believed it was a fundamental part of learning. However, P1 stated the recent students in the department were still not able to instil eye drops, but felt it was important for them to practise the technique; this highlighted the inconsistencies in different departments.

All participants who had some experience of assistance from play specialist (75%) stated that it was beneficial to the overall experience. However, P1 and P6 felt it was beneficial to specific patients only.

The study found that all of the departments sent generic letters advising eye drops may be instilled at the appointment; however, one department also used a video on their hospital website to explain what happens at the appointment. Most participants did not have experience of using videos but suggested it as a potential improvement.

Eye sprays have been used as a trial in the UK previously; they were mentioned as an improvement to process of instilling eye drops by one participant. Whilst none of the participants had any experience of using eye sprays which meant the efficacy was unknown, this subtheme was not discarded because it was still suggested as a potential improvement. This was relevant to the overall study and did not make this concept less important.

## Discussion

The study has highlighted several important aspects of the views and experiences of orthoptists that have gone unrecorded in previous literature. The instillation of eye drops in children is a form of practise which reveals deployment of highly sophisticated and nuanced communication, this highlighted ways in which restraint was conceptualised and articulated by orthoptists. Restraint has been widely researched in children undergoing invasive medical procedures such as cannulation (Bray et al., 2016; Svendsen et al., 2017) but not specifically eye drops. To address the difficulties of restraint, the orthoptist used positive language and asked for help from parents or colleagues. Even with sophistication of the process and the ways in which restraint was dealt with, the orthoptists made suggestions on how the practise could be developed to improve the overall process.

### Communication

The data emphasised the importance of verbal and non-verbal communication in a healthcare setting for any health professional and further explored the highly skilled techniques adopted by the orthoptists. Sim et al. (2009) claimed effective verbal communication by orthoptists with adult patients and verbal skills such as encouragement and repetition were a key aspect for compliance of treatment. Pilon et al. (2020) stated that children anticipated the eye drops to be worse than they were; this highlighted the importance of effective communication to ensure the child is as relaxed as possible prior to instillation. Our study showed that effective verbal communication took many forms, one form was the language used around the child when describing the eye drops. This was an interesting finding because eye drops were described as something positive and non-invasive. Mason and Stevens (2010) advised demonstration of the eye drops on the back of the child’s hand. This non-verbal technique enabled the child to visualise the eye drops as non-threatening and allowed them to frame the anticipated experience as something familiar, such as water.

The use of positive terminology regarding ‘restraint’ was apparent from the study and euphemisms were used, this is also reiterated by Svendsen et al. (2017) who suggested the term ‘restraint’ should not be used and replaced by terminology such as ‘holding’, similarly Mason and Stevens (2010) used holding in a ‘gentle, comforting manner’. This was consistent with our orthoptist’s accounts of using carefully chosen language when asking parents for help. The orthoptists stated that they asked parents to ‘cradle’ the child; this terminology has connotations of reassurance and safety and were used to make the parent and child feel comfortable. In contradiction to this, some orthoptists stated that they would inform the parents of the side effects and what is expected following instillation which would have likely took place in front of the child, this could alarm the child undoing the efforts to relax them.

### Adaptation of the process

Our data underscores the belief that each child was different with individual needs and that the process must be adapted. Orthoptists believed that explaining the procedure to the child with additional needs such as autism was essential. Being adaptable was important and therefore the development of guidelines were not suggested by any of the orthoptists. Bray et al. (2016) found that children who had experienced previous difficulties with procedures had information withheld in some instances and the child was not informed of what was about to happen. This was seen as in the child’s best interest; similarly, our orthoptists would have had to use discretion on the best practise for the child depending on their needs, in some cases alternatives such as home dilation were considered.

### Restraint

Our study explored the ways in which restraint was conceptualised and articulated by orthoptists. Svendsen et al. (2017) explored factors on how restraint was defined by professionals and how they reasoned about it during cannulation. Whilst the need for restraint was considered necessary, there was a general consensus that measures could be taken to reduce or minimise the need for restraint if the challenges were considered, such as the reaction of the parent. Svendsen et al. (2017) found the role of parents to be essential in minimising resistance from the child; this was similar to our findings. The orthoptists believed that help is required with physical restraint, and this is usually sought from the parents first. The involvement and reliability of parents was seen as an integral part of the process of eye drops instillation, this was someone the child trusted. In addition to this, the job of the parents was to soothe and relax the child. If this was unsuccessful or the child was uncooperative, help was sought from colleagues to help restrain the child. Asking parents to assist was beneficial as mentioned by Bray et al. (2016) who found that many healthcare professionals were uncertain about the boundaries of holding a child for a clinical procedure. Whist the reasoning behind orthoptists asking parents for help may differ from the reasons stated by Bray et al. (2016), parents helping with restraint was found to be an essential part of the process.

### Home dilation

As investigated by Law et al. (2020), the most common themes emerging from parents’ accounts of instilling eye drops were the techniques used, eye drops challenges and the role of healthcare professionals. Administration techniques included using force and restraint, the use of distraction techniques involving games, and waiting until the child was asleep before instilling the eye drops. Home dilation was suggested by our orthoptists as a technique to address the challenges of eye drops in clinic; however, the data did not go into the exact detail of how this would be carried out. We have assumed that it would involve parents instilling the eye drops at home.

### Play specialists

Hirji et al. (2012) found that there were multifactorial reasons for stress caused from the eye drops in children, not just the eye drop itself. Distress was worsened by longer waiting times and the use of bright lights. The study found that children under the age of five were more likely to be distressed, whereas children above the age of 8 tolerated eye drops well due to having developed coping mechanisms for pain and fear. Law et al. (2020) found that the most common challenges with instilling eye drops included distress for children and negative emotions for parents, such as anxiety. Our orthoptists highlighted how play specialists could develop practise; they could address the long waiting times and the bright lights and could help with keeping the child distracted by being present in the waiting and clinical rooms before and during the examination.

Mason and Stevens (2010) suggested asking someone for help with holding up a toy, which could be done by play specialists or parents. It is important to note that all the orthoptists who received assistance from play specialists found it beneficial, especially with specific patients. Whilst there was no mention of parents acting as a distractor in our study, McCarthy et al. (2010) found parent-provided distraction during intravenous insertion was a logical use of resources with training parents to become distraction coaches for their child, however some parents were more effective than others. The variability of the parents further supports our orthoptists’ accounts of having the assistance of a play specialist when required.

### Medical exemptions

Studying the medical exemptions module was a key recommendation on how the practise could be developed. This finding encourages qualified orthoptists to study the medical exemptions module as a stand-alone module to improve their technique and understanding of eye drops. One of the participants mentioned that they had learnt a new technique by applying eye drops on the lashes of older children for them to blink them in. The orthoptists in our study do not state a specific age; therefore, it could be attempted with younger children if cooperative. This technique was also recommended by Zurevinsky et al. (2016), who found that applying eye drops on the inner canthus whilst the eyelids were closed was likely to cause less distress to the child and that the technique dilated the pupils the same as forcefully opening the eyelid. There is an argument that the technique could be taught by colleagues without studying the module; however, the module delves into a deeper understanding of eye drops and other medications, and this knowledge can only be gained by studying the module. An interesting finding was that only some participants were involved with instilling eye drops as a student; this was dependent on the clinical placement site. Therefore, some students may never have had an opportunity to practise instillation prior to qualifying as an orthoptist.

### Eye sprays

The practise could be developed with the use of eye sprays, as suggested by a participant. This has been documented in previous literature, such as Syrimi et al. (2013), who measured distress in UK children by comparing eye drops and eye sprays and concluded that eye spray caused significantly less distress than eye drops. However, they found that the dilation of the pupils was not fully achieved with the eye sprays. In contrast, Chafai et al. (2006) found the efficacy of the eye sprays was just as effective as eye drops and also concluded that eye sprays caused less distress in children. More recently, Vagge et al. (2020) concluded there was significantly less distress from the child at the time of receiving eye sprays except in the age group of 6, which was the upper limit of the age criteria. However, almost 17% of children with dark irises did not dilate enough for refraction. Therefore, further investigations would be required to assess efficacy, practical improvement and cost-effectiveness, because none of the orthoptists had previous experience with eye sprays. However, if effective, it could revolutionise how orthoptists instil eye drops in children.

### Information videos

The process could be developed with information videos to make the child and parent feel more comfortable. One department also used a pre-procedural video to inform the parent and child of what is expected at the appointment to best prepare the child. Kolk et al. (1999) explored other invasive procedures such as venepuncture for blood tests in children and concluded that preparing children for blood tests with pre-procedural information was an important factor in reducing distress levels. This was consistent with Bray et al. (2016), who stated that some children were prepped at home and informed of the procedure prior to attending the hospital, as parents felt it was important for the child to know what was about to happen. Similarly, Pilon et al. (2020) found that whilst there was no significant difference in distress levels for children who received an information booklet and those who did not prior to the instillation of the eye drops, it did prove a positive factor for some children. Parents also preferred to continue with the booklet because they believed that this gave a sense of control to the child. This resulted in less restrictive holding of the child and a more positive experience. In our study, the information video about eye drops was only used by one department and recommended by another, despite it being a modern way of providing information. Videos with popular cartoon characters are currently used in other areas of healthcare, such as visiting the dentist or opticians, and therefore could be the future of preparing children for eye drops.

Orthoptists believed that compliance with treatment, such as wearing a patch at home, was not impacted by the instillation of eye drops (Table 1). Wallace et al. (2013) claimed compliance to treatment of lazy vision was affected by the lower attendances to the eye appointments and prolonged treatment but there was no mention from families about experience of eye drops in clinic affecting compliance in either study. This reflected our orthoptist’s accounts.

### Limitations

There were some limitations of the study; it was a small-scale research effort with a sample size of eight. However, this provided a significant amount of data. The non-random nature of the sample meant that the findings cannot be generalised, and certain views may not have been captured, though the participants had a range of experiences and were from eight different NHS Trusts across the UK. Some participants had worked at different hospitals, and two participants had also worked abroad in Australia. The participants had studied at different universities, such as Sheffield and Liverpool. We are confident that the findings in this study give an insight into the views and experiences of orthoptists and how practise can be developed. This study has taken a modest step towards addressing a gap in existing research.

## Conclusion

Knowledge in this area of research is limited and there is no prior investigation on the views and experiences of orthoptists instilling eye drops in children’s eyes. Our study has contributed to a comprehensive understanding of these views and experiences. The results portrayed a variety of key findings.

It is evident that the instillation of eye drops in children is a practise which reveals deployment of highly sophisticated and nuanced communication. The encounter consists of highly adopted skills, which take into consideration the individual needs of children. The study articulates and conceptualises how ‘restraint’ is viewed by orthoptists and how the requirement of help from parents is essential to build trust in the child and best prepare them for the eye drops.

The study concludes that orthoptists embrace the instillation of eye drops as an important and unavoidable part of their role. The execution of this task comprises a sophisticated professional intervention entailing interaction with patients, parents and colleagues through physical means and various forms of verbal and non-verbal communication, some of which are complex. We can also conclude play specialist support and training as an orthoptic student is variable depending on the Trust.

### Recommendations

Departments should consider the availability of a play specialist in eye clinics. Some of the orthoptists were able to seek support from a play specialist, which helped the overall process making the child feel more comfortable, though this support was only available in four departments. We understand that there is a financial impact on the employment of play specialists. However, their presence will be an improvement to the process of instilling eye drops and quality of care, especially if the Trust already employs play specialists in the paediatric department within the hospital.

Individual departments should develop pre-procedural information videos as used in other areas of healthcare to help prepare the child and parents for the eye drops. This was suggested as a potential improvement to the overall experience to familiarise children with the process. Other studies involving prepping children for blood tests or medical procedures have claimed that it reduces anxiety in the child if they are aware of the process prior to attending the appointment. But, further studies would be required to investigate the impact of pre-procedural information videos specifically on instillation of eye drops.

The study of the medical exemptions by orthoptists is recommended to improve technique and gain a deeper understanding of eye drops. For orthoptists who have qualified before the introduction of the medical exemptions module as part of the orthoptic degree, we recommend undertaking this module as part of a Masters or as a stand alone module. It was evident that some orthoptists felt that a new graduate orthoptist would be better prepared to deal with the challenges of instilling eye drops because they had undertaken this module along with practising instillation as a student. These highlighted further inconsistencies because some of the orthoptists were involved in instilling eye drops as a student and some were not; this varied in different departments. A more consistent approach to practising instilling eye drops as an orthoptic student across different placement sites is recommended.

Our study was an important area of research because there are currently no studies exploring the orthoptist perspective. In conclusion, orthoptists believed eye drops are a part of their role within a team of professionals looking after children’s eyes. The orthoptists recommended techniques to address the challenges with instilling eye drops, which will encourage other working orthoptists to reflect on their practise and adopt new methods. The orthoptists made suggestions on how the process of instilling eye drops in children can be improved, which could be adopted by Trusts. Whilst the study was small-scale, it could result in improvements and recommendations to current practises and influence change within other fields of medicine such as blood tests and MRI scans in children. Further studies could also investigate the parental perspective on how the instillation of eye drops in the eye clinic could be improved.

## Appendix 1: Topic guide

The interview will begin by checking that the interviewee is happy to proceed and by recapping the purposes of the study and the expected time for completion of the interview. The interview will cover the following topics:

- Background experience of the orthoptist. When did the Orthoptist qualify?
- The experiences of orthoptists of instilling eye drops in children attending the eye clinic and how these experiences are viewed by orthoptists (to explore common aspects of these experiences).
- The challenges orthoptists have come across when instilling eye drops in children.
- Are there any specific examples of good/bad experiences?
- At what point does the orthoptist ask colleagues for help?
- When does the orthoptist decide against instilling eye drops?
- Has the orthoptist ever been injured during the instillation of eye drops?
- How has this affected the orthoptist?
- Orthoptist experience of how the patient/clinician relationship is affected.
- Orthoptist experience as to what works well when instilling eye drops in children.
- Orthoptist experiences with distraction techniques.
- Orthoptist experience with using eye sprays or anaesthetic eye drops before the dilation eye drop.
- Orthoptist experience with different techniques with children experiencing distress, hesitancy or special needs.
- Orthoptist suggestions for improvements to the experience of instilling eye drops for the child and/or orthoptist. What more could be done to help improve the experience for patients and orthoptists? Is further training required?
- Any further observations/remarks the interviewee would like to make.
